# The evolution of Taiwan’s National Health Insurance drug reimbursement scheme

**DOI:** 10.1186/s40199-014-0080-7

**Published:** 2015-02-10

**Authors:** Jason C Hsu, Christine Y Lu

**Affiliations:** School of Pharmacy and Institute of Clinical Pharmacy and Pharmaceutical Sciences, College of Medicine, National Cheng Kung University, No.1, Daxue Rd., East Dist., Tainan City, 70101 Taiwan R.O.C; Department of Population Medicine, Harvard Medical School and Harvard Pilgrim Health Care Institute, Boston, MA USA

**Keywords:** Universal health coverage, Drug policy, Reimbursement, Medicines coverage, National Health Insurance, Taiwan

## Abstract

**Background:**

The rapid growth of health care expenditures, especially pharmaceutical spending, is a challenge for many countries. To control increasing pharmaceutical expenditures and to enhance rational use of drugs, Taiwan’s National Health Insurance drug reimbursement system has evolved over time since its introduction in 1995. This study reviewed Taiwan’s drug reimbursement scheme: its development and evolution in the last two decades, and implications and impacts of recent policies for drug pricing. We also provide recommendations for possible improvement.

**Methods:**

We conducted a review of Taiwan’s National Health Insurance drug reimbursement scheme. We focused on three major components of the scheme: (i) the scope of drug coverage; (ii) pricing system for pharmaceuticals under the scheme; and (iii) adjustment of drug reimbursement prices. We reviewed the literature and public policy documents.

**Results:**

The National Health Insurance delisted 176 and another 240 behind-the-counter products (e.g., antacids, vitamins) between 2005 and 2006 to reduce pharmaceutical expenditures. For the pricing of pharmaceuticals, policy evolution can be divided into four phases since 1995; the present system emphasizes stakeholder engagement, health technology assessment, domestic R&D, and improving quality of products. To close the gap between drug reimbursement prices and procurement prices, eight rounds of drug price surveys and adjustments have been implemented since 2000.

**Conclusions:**

Taiwan’s National Health Insurance drug reimbursement scheme has evolved substantially over time to provide more equitable and affordable access to prescription medicines. However, more work is still needed as irrational difference in reimbursement and procurement prices persists and the total expenditure of the drug reimbursement scheme continues to increase at unsustainable rates.

## Introduction

Access to health care, including ‘essential’ medicines, is regarded as a human right by the International Covenant on Economic, Social and Cultural Rights [1]. The vast majority of people and governments around the world generally support the implementation of national health insurance. Many economically developed countries have implemented national health insurance that aims to provide its members with satisfactory care services to achieve “health for all” [2]. However, many healthcare systems have sometimes achieved poor performance given the resources spent and/or are still undergoing reform.

In Taiwan, the National Health Insurance (NHI) is a compulsory social insurance system in which the coverage rate of its 23 million residents is as high as 99% currently. Since the introduction in August 1995, the National Health Insurance has gained public recognition as Taiwan becomes comparable with neighboring countries (like Japan, South Korea and Singapore) in terms of quality of care, healthcare cost control, drug spending growth, and public satisfaction [3]. However, concerns have been raised about its financial sustainability.

Around the world, countries generally adopt a pluralistic system of healthcare coverage that maximizes consumer choice (e.g., USA) or a predominantly single, universal scheme for healthcare coverage that maximizes equity and the prospects for cost control. Taiwan adopts the single system model. The National Health Insurance is contracted with public, private, and corporate healthcare institutions, which provide a range of covered healthcare services, including prescription drugs. The National Health Insurance Administration (NHIA; formerly known as the Bureau of NHI, the name was changed in 2013), set up by the government, reimburses the contracted institutions for the services provided.

**Figure 1:**
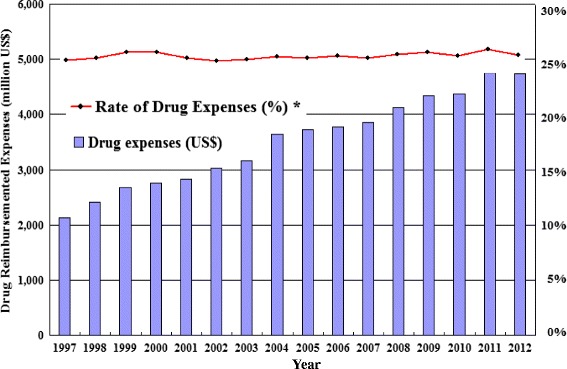
**The evolution of drug reimbursement expenses.** *Rate of Drug Expenses (%): proportion of drug expenditure of total health care costs.

The rapid growth of healthcare costs is a challenge faced by all countries, especially the growth of pharmaceutical costs, which is even more evident [4,5]. The total drug expenditure of Taiwan’s National Health Insurance was about US$2,133 million in 1997, and it increased by around US$173 million each year. In 2012, pharmaceutical expenditures reached US$4,733 million, which accounted for 25.1% of the total healthcare expenditure (Figure 1) [3]. The main causes of rising healthcare costs and pharmaceutical expenditures include the aging population, the increasing number of patients with chronic diseases, increasing drug prices, larger drug usages, and the availability of new, more expensive drugs [6-8].

To control growing pharmaceutical expenditures, the NHIA implemented multiple policies for prescription drug reimbursement. The purpose of this study was to review and summarize the evolution of Taiwan’s drug reimbursement scheme over the last two decades, including its development and major changes for drug pricing, and implications and impacts of its recent policies. We also highlighted possible policy-induced problems that need to be addressed. Finally, we provide some recommendations for how Taiwan’s drug reimbursement scheme can continue to evolve to ensure the goals of financial sustainability and rational use of medicines [9].

## Method

We conducted a review of Taiwan’s National Health Insurance drug reimbursement scheme. We focused on policies implemented by the NHIA over the last two decades. We reviewed policies that targeted different issues: (1) the scope of drug coverage, (2) the pricing system for pharmaceuticals under the scheme, and (3) adjustments of drug reimbursement prices. Similar to many countries, medicines are classified into three categories in Taiwan: (a) prescription drugs, (b) drugs designated by physicians or pharmacists, which can be purchased at pharmacies without prescriptions (e.g., antihistamines, antitussive agents) – generally known as behind-the-counter or pharmacist only drugs, and (c) over-the-counter (OTC) medications.

We collected and reviewed historical archives including official documents, books, published articles, research projects, conference records, websites, newspapers, speeches etc. After reviewing abovementioned materials relating to the drug reimbursement scheme, we also examined policy implementation and policy changes, summarized the known impacts of the policies, and highlighted possible policy-induced problems that need to be addressed for system improvement.

## Results

NHIA policies over the last two decades largely targeted prescription drugs and behind-the-counter drugs.

### Policies governing the scope of drug coverage

According to Article 51 in the National Health Insurance Act (amended in 2011) [10], the NHIA does not reimburse the following: (1) medicines that are approved by the Taiwan Food and Drug Administration (Taiwan FDA) but are not used for disease treatment, such as contraceptive, hair tonic, dark spots detergent, smoking cessation patches; (2) some vaccines (e.g., quadrivalent Human papillomavirus types vaccine); (3) over-the-counter drugs and non-prescription drugs which should be used under the guidance of a physician or pharmacist (also called the behind-the-counter drugs); (4) drugs for human-subject clinical trials; (5) drugs which are deemed by the National Health Insurance as not essential for medical treatment (e.g. dentures, artificial eyes, spectacles, hearing aids, wheelchairs, canes, and other treatment equipment) or not cost-effective; (6) drugs which do not conform to the indication that stipulated in the approved indication for licensing and/or the “Reimbursement Restriction” enacted by the National Health Insurance. However, in special cases, an application for prior authorization can be made to the National Health Insurance, and the drug will be reimbursed if authorization is given; and (7) any other drug which the NHIA publicly announces that it will not be reimbursed [11].

Behind-the-counter drugs were covered by the former Civil Servant Insurance and Labor Insurance and in the early years of the National Health Insurance. The NHIA reviewed and reduced the scope of coverage of behind-the-counter drugs over time [11]. Some behind-the-counter drugs were delisted to meet the priorities of the National Health Insurance, in accordance with Article 51 of the National Health Insurance Act, and more generally, to establish patient expectations and the culture of rational use of medicines and basic self-healthcare. This change also intended to reduce costs of the National Health Insurance as well as making better use of its resources for treatment of major diseases.

This delisting of behind-the-counter drugs also benefited consumers who needed such products for treatment of minor illnesses (e.g., headache, cold). They can save time and related expenses when they choose to purchase medicines at pharmacies instead of visiting physicians at primary care clinics or hospitals. For example, a patient’s out-of-pocket costs are less if s/he chooses to purchase medicines for headache from pharmacies (only US$3) compared with visiting a physician which requires physician visit copays (US$5).

In total, the National Health Insurance delisted 176 (e.g., some antacids) and 240 (e.g., some vitamins, electrolytes) behind-the-counter products in 2005 and 2006 respectively. However, there was considerable resistance from both physicians and patients; thus, no further behind-the-counter drugs were delisted. Currently, there are still around 1400 behind-the-counter products reimbursed by NHIA (e.g., gastrointestinal drugs, antihistamines, antitussive agents) [3]. However, delisting in the future is likely under the pressure to contain costs.

### The pricing system for pharmaceuticals under the drug reimbursement scheme

**Figure 2:**
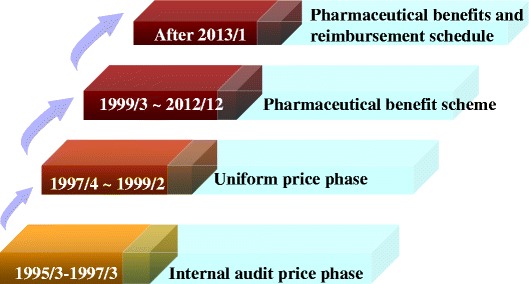
**The evolution of drug pricing of the drug reimbursement scheme under Taiwan’s National Health Insurance.**

The pricing system under the drug reimbursement scheme of the National Health Insurance can be divided into four phases over time: (1) internal audit price (1995/3-1997/3); (2) uniform pricing (1997/4-1999/2); (3) Pharmaceutical benefit scheme (1999/3-2012/12); and (4) Pharmaceutical benefits and reimbursement schedule (after 2013/1), which are described as below and summarized in Figure 2.

Prior to the implementation of the National Health Insurance in 1995 in Taiwan, about 50% of the population was insured under the Civil Servant Insurance, Labor Insurance, and Farmer’s Health Insurance. At the time, pharmaceutical companies were allowed free pricing, and they were subject to hospitals’ pharmaceutical tender and negotiation to determine the price of drugs in hospitals. Hospitals would bill the insurers. The insurers would then reimburse individual hospitals by an approach known as “transaction cost-plus”. For drug reimbursements, the joint bid price would be paid to public hospitals while to this price plus 10-20% would be paid to private hospitals. Profits were usually used to pay for drug warehouse management, dispensing and other expenses. High-level hospitals tended to use more expensive, brand-name drugs or imported drugs because of profits from pharmaceutical sales. At the time of public bidding in public hospitals, manufacturers were reluctant to cut prices, resulting in high tender prices. Prescription drugs were paid out-of-pocket in primary care settings because most patients were not insured under the Government Employee’s Insurance, Laborer Insurance, or Farmer’s Health Insurance. As a result, patients were sensitive to drug prices; many would choose domestic, generic drugs over the more expensive, brand-name drugs or imported drugs.

At the early stage of the National Health Insurance (1995–1997), the NHIA released the “National Health Insurance Drug Items Table” that listed products being covered, and drugs were reimbursed through the “internal audit pricing” approach. However, the internal audit pricing system was unclear, and the price of imported generic drugs was relatively high due to a lack of international drug price comparison. These led to substantial variations in drug prices between domestic drugs and drugs by international manufacturers [12]. Hospitals generally adopted the “fee for service” approach for drug reimbursements while primary care clinics adopted the “fixed fees by days of supply” approach (e.g., one-day supply of any medications received a reimbursement of NT$35, regardless therapeutic indication and actual procurement price; two-day supply was NT$70; and three-day supply was NT$100). The following ‘consequences’ were observed: (1) primary care clinics tended to reduce drug costs and were reluctant to release prescriptions to patients so patients had to obtain drugs at the clinics (resulting in the phenomenon of “next-door pharmacy”, which had negative impacts on the separation of drug prescribing and dispensing); (2) some patients were transferred to higher level hospitals in order to obtain drugs of higher prices and/or for longer supplies; and (3) use of drugs of higher prices in primary care clinics was subsequently reduced [13,14].

During 1997–1999, the NHIA invited the pharmaceutical industry to engage in the development of pricing guidance for drugs covered under the NHI (“National Health Insurance drug pricing principles”). The goals were to lower drug prices, control the growth of drug prices, reduce the prices of brand-name drugs and generic drugs, encourage the use of generic drugs, and protect the domestic generic drug market. The pricing guidance governed drug pricing by a drug classification system: (1) new drugs: the NHIA invited the medical and pharmaceutical experts to engage in the review and approval process; (2) the compound and special specification drugs: paid the same minimum price as other drugs of the same composition; (3) brand-name drugs: brand-name drugs were subdivided into two categories: ones that have no bioavailability/bioequivalence (BA/BE) generic drugs as alternatives in the market, and others that have BA/BE generic drugs as alternatives. Drugs of the former category were priced according to the international drugs with average market prices; while the price of the latter category must not exceed 85% of the average market price of international drugs; (4) the price of BA/BE generic drugs must not exceed the price of brand-name drugs; and (5) the price of non-BA/BE generic drugs must not exceed 80% of the price of brand-name drugs. From then on, drugs covered by the National Health Insurance were uniformly priced.

During 1999–2012, the NHIA attempted to address the problem of differences between drug procurement and reimbursement prices. To determine the market drug price difference, the NHIA required hospitals and manufacturers to provide the actual transaction prices and trading volumes. However, many hospitals and manufacturers resisted such investigation or supplied false declarations about prices, and the drug price gap remained a serious problem. The NHIA therefore announced the “Pharmaceutical Benefits Scheme” in March 1999 to govern the listing of drugs, the pricing of pharmaceuticals, and the adjustment of drug reimbursement prices. It also set the target to reduce the gap in drug prices to less than 15% within five years. Moreover, NHIA announced in April 1999 that drug price surveys for NHI reimbursed drugs were to be conducted every 1–2 years. Since then, the NHIA has implemented eight drug price surveys; new drug reimbursement prices were announced and implemented respectively on April 1^st^, 2000, April 1^st^, 2001, March 1^st^, 2003, September 1^st^, 2005, November 1^st^, 2006, October 1^st^, 2009, November 1^st^, 2011 and May 1^st^, 2014 (Table 1) [15]. Adjustments of drug prices are discussed in detail in the next section.

**Table 1:** **List of drug price surveys and adjustments**

**Order**	**Date**	**Estimated cost-savings (million US$)**
1^st^	April 1^st^, 2000	16.67
2^nd^	April 1^st^, 2001	153.33
3^rd^	March 1^st^, 2003	190.00
4^th^	September 1^st^, 2005	81.00
5^th^	November 1^st^, 2006	500.00
6^th^	October 1^st^, 2009	195.67
7^th^	November 1^st^, 2011	--
8^th^	May 1^st^, 2014	--

Resource: Huang [15].

The “Pharmaceutical Benefit Scheme” was replaced by “Pharmaceutical benefits and reimbursement schedule” on January 1^st^, 2013. Compared with “Pharmaceutical Benefit Scheme”, the present new scheme emphasizes stakeholder engagement (including the insurer and the relevant authorities, experts and scholars, the insured, employers, health care service providers, etc.) to discuss and design the listing of drugs and reimbursement prices for specific products. Further, health technology assessment (considering human health, medical ethics, cost-effectiveness of products, and financial sustainability of the NHI) was required for new drugs prior to listing in the National Health Insurance under this new phase. In 2007, the Center for Drug Evaluation (CDE) was created to conduct health technology assessment (HTA), that is, assessment of comparative efficacy, cost-effectiveness, and budget impact of new drugs. The CDE only provides HTA reports to the NHIA, it is not involved in pricing. The NHIA considers HTA evidence as part of the information used for listing and reimbursement decisions [16].

### The adjustment of drug reimbursement prices

To ensure reasonable drug prices and close the gap between procurement and insurer reimbursement prices for prescription drugs, Taiwan made multiple efforts. Because institutions procure large quantities of medicines, procurement prices are typically lower than the amount reimbursed by the NHIA and the differences constitutes a profit for hospitals [17]. To assess procurement prices, the NHIA conducted surveys to obtain drug wholesale prices from pharmaceutical companies and procurement prices from hospitals since 1999 [18]. Reimbursements were adjusted if there was a difference of 30% or more between the average procurement price and the NHI reimbursed price. Prices were subsequently monitored and adjusted every two years for patented products, for products whose patent right has expired for more than five years, and for products that have no patent right. These drugs are further divided into the following two categories: (1) drugs from original R&D pharmaceutical companies, drugs of which the process of pharmaceutical form complies with “The Pharmaceutical Inspection Convention and Pharmaceutical Inspection Co-operation Scheme - Good manufacturing practice (PIC/S GMP)” requirements, BA/BE generic drugs, drugs approved to by the US FDA and/or The European Medicines Agency for marketing, controlled items of BE generic drugs; (2) common generic drugs which do not fall into the first category [11].

Studies have been conducted to examine effects of drug reimbursement price reductions in Taiwan. Lee et al. [19] assessed the effects of six drug price policies and found that they reduced pharmaceutical expenditures, especially for outpatient medications and for hospitals (compared with clinics) [19]. Chen et al. [17] showed that reimbursement price reductions for targeted cardiovascular medications reduced the daily medical use and expenditures, but did not affect non-targeted products [17]. Chu et al. [20] studied price reductions for anti-hypertensive drugs. They suggested that reimbursement price adjustments may have created an incentive for physicians to prescribe drugs with higher profit margins, and to increase prescription duration or the number of drug items per prescription [20]. Hsiao et al. [21] did not find strong associations between reimbursement price adjustments and drug utilization and expenditures during 2001–2004. Chu et al. [22] studied effects of reimbursement price adjustments on outpatient hypertension treatments among the elderly. They found that the average cost per prescription increased slightly, and that physicians tended to prescribe drugs whose prices were not reduced instead of those subject to price reductions. Findings by Hsu et al. [18] indicated that prescribing shifted from targeted to non-targeted products [18]. Overall, these studies suggest shifts in use from targeted to non-targeted products to maintain profits from drug price gaps but whether they reduced pharmaceutical expenditures is unclear.

## Discussion

The study provides a review of the development and evolution of Taiwan’s universal drug reimbursement scheme under its National Health Insurance. We highlighted major policy changes for drug pricing over the last two decades and their known impacts and implications. It is important to note that many policy changes (e.g., delisting of behind-the-counter products, introduction of HTA) remain to be evaluated for their impacts on medication use, drug prices, quality of care, and pharmaceutical expenditures.

With the goal to reduce pharmaceutical expenditures to the government, about 400 behind-the-counter products were delisted from Taiwan’s national Drug Reimbursement Scheme. This coverage change also aimed to establish patient expectations and the culture of rational use of medicines and basic self-healthcare for minor illnesses such as headache or cold. This is not surprising; many national drug coverage schemes do not reimburse or reimburse only selected few behind-the-counter products, e.g., Australia’s Pharmaceutical Benefits Scheme. Misuse, under-use or over-use of behind-the-counter drugs are possible unintended consequences. Inappropriate consumer self-medication may lead to subsequent medication-related adverse health outcomes (e.g., medication error or poisoning), or negative health outcomes if appropriate treatment was delayed or not used, which may lead to subsequent increase in healthcare costs. It is important that delisting of behind-the-counter medications is accompanied by appropriate educational programs directed to consumers and pharmacists to ensure rational use of medicines. Taiwan may learn from Australia, which has worldly recognized multifaceted programs to improve rational use of medicines [23].

To ensure reasonable drug prices and close the gap between procurement and insurer reimbursement prices for prescription drugs, Taiwan and other countries (e.g., China) [24] have made multiple efforts. We highlighted how the pricing system under Taiwan’s Drug Reimbursement Scheme has evolved over time. While the gap between drug procurement and reimbursement prices has narrowed, price difference persists. For instance, there are substantial price difference between drug reimbursements and claims made by primary care clinics for prescription drugs that use the “fixed fees by days of supply” approach mentioned above.

Whether drug price adjustments achieved the intended cost-savings is unclear. Despite several waves of drug price adjustments to close the gap between procurement and reimbursement prices, the total pharmaceutical expenditures is still on the rise (average yearly growth rate for pharmaceutical expenditures from 1997 to 2012 is 8.13%). Reasons for such growth include: (1) adjustments of drug reimbursement prices only reduce the prices of targeted products, not the volume of use; (2) off-label use: it has not been estimated how much expenditures are attributed to use of prescription medications outside approved indications under the Drug Reimbursement Scheme; reimbursed indications are largely based on clinical treatment guidelines, Taiwan FDA approved indications and specification made by NHIA or medical associations; (3) drug waste (unnecessary use and stockpiling of medications): this issue is particularly common for behind-the-counter drugs (e.g., antacid agents and vitamins), which led to delisting of some products by the NHI; however, delisting was opposed by many physicians and patients; and (4) the availability of innovative, expensive drugs such as cancer targeted therapies: the NHI created the HTA body to assess the cost-effectiveness of new drugs to inform decisions about reimbursement.

We recommend that capitation/case payment models [25], diagnosis related groups (DRGs) [26-28] and pay for performance [29,30] are some possible alternative approaches, that have been used by other countries and show promise in controlling total pharmaceutical expenditures without substantially reducing the quality of health care.

Health Technology Assessment is increasingly adopted for making drug reimbursement decisions throughout the Asia-Pacific markets. Apart from Taiwan, countries in this region with established HTA system include Australia, New Zealand, South Korea, and Thailand [31,32]. Economic evaluation should not only inform the decision of reimbursement but also used to negotiate prices with manufacturers. In addition, many countries (including countries in Asia-Pacific markets such as Australia, South Korea) are adopting risk-sharing agreements for funding high-cost innovative drugs such as adalimumab and imatinib. Risk-sharing agreements are typically between a payer and a pharmaceutical company in which the partners negotiate the price of a product and/or the overall spending depending on volumes sold, clinical outcomes achieved or patient populations who receive the drug [33-35]. The intent is that companies share the financial risk of payers to reimburse the drug, and pay for the drug when an agreed volume or budget is exceeded, or intended clinical outcomes are not achieved. Taiwan could learn lessons from neighboring countries adopting HTA and risk-sharing agreements to address the challenge of high-cost medicines.

## Conclusion

Taiwan’s Drug Reimbursement Scheme under its universal National Health Insurance has come a long way over the last two decades. It is highly regarded particularly on the basis of comprehensive drug coverage, minimal patient cost burden, and timely access to new medicines. The NHI implemented multiple policy changes to enhance rational use of drugs and to contain increasing pharmaceutical expenditures. However, while the data are limited, there were opposition from consumers and physicians for some of the changes. Many policy changes remain to be evaluated for their impacts on medication use, quality of care, and pharmaceutical expenditures. Further policy changes may be needed and these should be developed in light of lessons learned by other countries that are also facing similar challenges. Stakeholders (i.e., patients, clinicians, government, industry) need to work closely together to continue to improve rational use of drugs, the quality of healthcare, and the financial sustainability of the National Health Insurance. Evidence-informed policy changes with appropriate stakeholder engagement will be important for optimal patient outcomes.

## Abbreviations

R&D: Research and development
NHI: National Health Insurance
NHIA: The National Health Insurance Administration
OTC: Over-the-counter
Taiwan FDA: Taiwan Food and Drug Administration
BA/BE: Bioavailability/bioequivalence
CDE: Center for Drug Evaluation
PIC/S GMP: The Pharmaceutical Inspection Convention and Pharmaceutical Inspection Co-operation Scheme - Good manufacturing practice
DRGs: Diagnosis related groups
HTA: Health technology assessment
